# Informative cluster size in cluster-randomised trials: A case study from the TRIGGER trial

**DOI:** 10.1177/17407745231186094

**Published:** 2023-07-13

**Authors:** Brennan C Kahan, Fan Li, Bryan Blette, Vipul Jairath, Andrew Copas, Michael Harhay

**Affiliations:** 1 MRC Clinical Trials Unit at UCL, London, UK; 2 Department of Biostatistics, Yale School of Public Health, Yale University, New Haven, CT, USA; 3 Department of Biostatistics, Epidemiology & Informatics, Perelman School of Medicine, University of Pennsylvania, Philadelphia, PA, USA; 4 Division of Gastroenterology, Department of Medicine, Schulich School of Medicine & Dentistry, Western University, London, ON, Canada; 5 Department of Epidemiology and Biostatistics, Western University, London, ON, Canada

**Keywords:** Cluster-randomised trial, estimand, informative cluster size, participant-average treatment effect, cluster-average treatment effect

## Abstract

**Background:**

Recent work has shown that cluster-randomised trials can estimate two distinct estimands: the participant-average and cluster-average treatment effects. These can differ when participant outcomes or the treatment effect depends on the cluster size (termed informative cluster size). In this case, estimators that target one estimand (such as the analysis of unweighted cluster-level summaries, which targets the cluster-average effect) may be biased for the other. Furthermore, commonly used estimators such as mixed-effects models or generalised estimating equations with an exchangeable correlation structure can be biased for both estimands. However, there has been little empirical research into whether informative cluster size is likely to occur in practice.

**Method:**

We re-analysed a cluster-randomised trial comparing two different thresholds for red blood cell transfusion in patients with acute upper gastrointestinal bleeding to explore whether estimates for the participant- and cluster-average effects differed, to provide empirical evidence for whether informative cluster size may be present. For each outcome, we first estimated a participant-average effect using independence estimating equations, which are unbiased under informative cluster size. We then compared this to two further methods: (1) a cluster-average effect estimated using either weighted independence estimating equations or unweighted cluster-level summaries, and (2) estimates from a mixed-effects model or generalised estimating equations with an exchangeable correlation structure. We then performed a small simulation study to evaluate whether observed differences between cluster- and participant-average estimates were likely to occur even if no informative cluster size was present.

**Results:**

For most outcomes, treatment effect estimates from different methods were similar. However, differences of >10% occurred between participant- and cluster-average estimates for 5 of 17 outcomes (29%). We also observed several notable differences between estimates from mixed-effects models or generalised estimating equations with an exchangeable correlation structure and those based on independence estimating equations. For example, for the EQ-5D VAS score, the independence estimating equation estimate of the participant-average difference was 4.15 (95% confidence interval: −3.37 to 11.66), compared with 2.84 (95% confidence interval: −7.37 to 13.04) for the cluster-average independence estimating equation estimate, and 3.23 (95% confidence interval: −6.70 to 13.16) from a mixed-effects model. Similarly, for thromboembolic/ischaemic events, the independence estimating equation estimate for the participant-average odds ratio was 0.43 (95% confidence interval: 0.07 to 2.48), compared with 0.33 (95% confidence interval: 0.06 to 1.77) from the cluster-average estimator.

**Conclusion:**

In this re-analysis, we found that estimates from the various approaches could differ, which may be due to the presence of informative cluster size. Careful consideration of the estimand and the plausibility of assumptions underpinning each estimator can help ensure an appropriate analysis methods are used. Independence estimating equations and the analysis of cluster-level summaries (with appropriate weighting for each to correspond to either the participant-average or cluster-average treatment effect) are a desirable choice when informative cluster size is deemed possible, due to their unbiasedness in this setting.

## Background

Cluster-randomised trials (CRTs) involve randomising groups of participants (such as hospitals or schools) to different treatment arms.^1–4^ Participants from the same cluster tend to be correlated (i.e. their outcomes are more similar to participants in the same cluster than to outcomes from participants in different clusters), and this correlation must be taken into account during analysis to obtain valid standard errors.^1–5^ Standard methods for analysing CRTs include mixed-effects models and generalised estimating equations (GEEs), while the analysis of cluster-level summaries (where the mean outcome is calculated for each cluster and the analysis is performed on these summaries) is often recommended when the number of clusters is small.^1–7^

However, there is growing recognition that these different estimators are estimating fundamentally different treatment effects in certain situations.^8,9^ We have recently shown that analyses of CRTs can estimate two different estimands: the participant-average treatment effect and the cluster-average treatment effect^8^ (we also need to select other aspects of the estimand, such as the strategies used to handle intercurrent events, whether the estimand is marginal or cluster-specific, and so on, but these choices are not the focus of this article). The key difference between the participant- and cluster-average estimands is the way the data are weighted. Specifically, the participant-average effect assigns equal weight to participants, while the cluster-average effect assigns equal weight to clusters. Thus, the participant-average effect provides the average effect across participants, while the cluster-average effect provides the average effect across clusters.^8^ Therefore, participant-average effects are most useful when interest lies in the intervention’s effect across participants, whereas cluster-average effects will be most useful when interest lies in the intervention’s effect across clusters (for instance, how the intervention modifies cluster-level behaviour).^8^ The value of these two estimands can differ when there is informative cluster size, which means that outcomes and/or treatment effects differ according to the cluster size (i.e. number of participants in the cluster) (see Table 1).^8,10,11^ When informative cluster size is present, estimators which target the cluster-average effect (such as the analysis of unweighted cluster-level summaries) will be biased for the participant-average effect, and vice versa.^8^ Furthermore, commonly used estimators such as mixed-effects models or GEEs with an exchangeable correlation structure may be biased for both the participant-average and cluster-average treatment effects.^8^ This is because the weighting used in these estimators is chosen based on efficiency, and thus corresponds to neither the participant- or cluster-average effects, but instead depends on both the cluster size and the intraclass correlation coefficient (the degree of correlation between participants in the same cluster), implying these models will incorrectly upweight treatment effects from certain clusters while down weighting effects from other clusters.^8,9^

**Table 1.** Summary of key concepts.

Concept	Description
Estimand	A precise description of the treatment effect investigators aim to estimate from the trial. Complete specification of the estimand requires defining the five core attributes^12,13^ (1) population, (2) treatment conditions, (3) endpoint, (4) summary measure and (5) handling of intercurrent events. For CRTs, additional elements also need to be specified, including whether the participant-average or cluster-average treatment effects are of interest (see definition below), as well as whether treatment effects are marginal or cluster-specific.
Participant- versus cluster-average treatment effects	In CRTs, two distinct estimands which may be of interest are the participant-average and cluster-average treatment effects. The key difference between the two estimands is how they weight the data: the participant-average effect gives equal weight to each participant (i.e. the average effect across participants), while the cluster-average effect gives equal weight to each cluster (i.e. the average effect across clusters).
Informative cluster size	Occurs when either outcomes or treatment effects depend on the number of participants in a cluster (e.g. if larger clusters have better outcomes/larger treatment effects than smaller clusters). For collapsible effect measures (e.g. a difference), the participant-average and cluster-average effects will coincide unless the treatment effect varies by cluster size; for noncollapsible measures (e.g. an OR), the two estimands will coincide unless outcomes or treatment effects vary by cluster size.
Estimator	The method used to analyse the data and compute an estimate of the treatment effect. Different estimators target different estimands, and some estimators are only unbiased if there is no informative cluster size.
Analysis of cluster-level summaries	A summary measure is calculated in each cluster (e.g. the mean), and a regression model is applied to the cluster-level summaries. Analyses can be either unweighted or weighted. Unweighted analyses estimate the cluster-average effect; weighted analyses (with weights equal to the number of participants in each cluster) estimate the participant-average effect. Analysis of cluster-level summaries is unbiased for both the cluster- and participant-average effects, regardless of whether there is informative cluster size.
IEEs	IEEs are applied to individual participant data and use an independence working correlation structure in conjunction with cluster-robust standard errors. They can be implemented using GEEs with an independence working correlation structure or using linear or logistic regression models provided a cluster-robust standard error is used. Unweighted IEEs estimate the participant-average effect. IEEs can also be weighted by the inverse of the cluster size to estimate the cluster-average effect. IEEs are unbiased for both the cluster- and participant-average effects, regardless of whether there is informative cluster size.
Mixed-effects models	Mixed-effects models are applied to individual participant data and include a random intercept for cluster. If there is no informative cluster size, the participant-average and cluster-average effects will coincide, and mixed-effects models target this common treatment effect.^14^ However, they can be biased for both the participant- and cluster-average estimand in the presence of informative cluster size.
GEEs with an exchangeable correlation structure	GEEs are applied to individual participant data, and an exchangeable working correlation structure is specified in conjunction with cluster-robust standard errors. If there is no informative cluster size, the participant-average and cluster-average effects will coincide, and GEEs with an exchangeable correlation structure models target this common treatment effect. However, they can be biased for both the participant- and cluster-average estimand in the presence of informative cluster size.^8,9^

CRTs: cluster-randomised trials; IEEs: independence estimating equations; GEEs: generalised estimating equations; OR: odds ratio.

An alternative estimation approach, which is unbiased even in the presence of informative cluster size, is the use of independence estimating equations (IEEs).^8–11,15–18^ IEEs are a class of estimator which use a working independence correlation structure in conjunction with cluster-robust standard errors. The working independence correlation structure ensures consistent estimation of the desired estimand,^8^ while the cluster-robust standard error corrects the standard error for the correlation within clusters.^19^ IEEs can be used to estimate both the participant- and cluster-average treatment effects, depending on how they are weighted (see Table 1).^8^ In addition, cluster-level summaries can also provide unbiased estimation of both effects, provided they are weighted appropriately (Table 1).^8^

However, in the absence of informative cluster size, IEEs and cluster-level summaries are likely to be less efficient than mixed-effects models and GEEs with an exchangeable correlation structure due to the working independence assumption.^20^ Typically, mixed-effects models and GEEs are only biased when there is informative cluster size, and to our knowledge, there have been no documented cases in the literature of informative cluster size occurring in CRTs. As such, trialists may be reluctant to move to potentially less efficient methods, such as IEEs and cluster-level summaries, without documented evidence that informative cluster size can occur in practice. However, to our knowledge, the presence of informative cluster size has never been formally explored in CRTs, which may explain its lack of documentation. The purpose of this article is to, therefore, perform a re-analysis of a published CRT to explore whether informative cluster size may be present.

## Methods

### The TRIGGER trial

Transfusion in Gastrointestinal Bleeding Trial (TRIGGER) was a CRT that compared two different thresholds for red blood cell transfusion (restrictive threshold versus liberal threshold) in patients with acute upper gastrointestinal bleeding.^21^ There were six hospitals (which acted as clusters), with the number of participants in each cluster ranging between 91 and 201. In TRIGGER, the scientific interest lay in the marginal participant-average treatment effect, that is, the average effect if all patients were assigned to the restrictive threshold versus if they were assigned to the liberal threshold. This estimand was of interest because the marginal participant-average effect provides the population-level impact of moving from one transfusion strategy to another. Further discussion on when participant- versus cluster-average effects and marginal versus cluster-specific effects will be of interest is available elsewhere.^8,22^

We re-analysed clinical outcome and adherence measures to compare to what extent estimates for the participant- and cluster-average effects differed. If estimates for the two effects differ, this may imply the presence of informative cluster size, which means that choosing an inappropriate estimator (i.e. one that targets the wrong estimand, or that relies on the assumption of no informative cluster size) could lead to bias.

We analysed 17 outcomes in total. Binary outcomes were further bleeding, thromboembolic or ischaemic events, and infection (each measured both in-hospital and up to day 28), as well as mortality, acute transfusion reactions, surgery/radiology, therapeutic endoscopy, whether the patient received at least one red blood cell transfusion, and full adherence to protocol (each measured in-hospital only). Continuous outcomes were the number of days spent in hospital, the number of red blood cell transfusions, average adherence (the percentage of Hb readings where the protocol was correctly followed), the EQ-5D score and the EuroQol-5D Visual Analogue Scale (EQ-5D VAS) score.

We initially planned to analyse mortality both in-hospital and at day 28, but found that results were identical between the two, and so only report in-hospital results.

### Methods of estimating treatment effects

For continuous outcomes, we estimated a difference in means, and for binary outcomes, we estimated a marginal odds ratio (OR). For each outcome, we implemented three different methods of estimation. The first targeted the participant-average effect using IEEs. The second targeted the cluster-average effect using either unweighted cluster-level summaries (for continuous outcomes) or weighted IEEs (for binary outcomes, to estimate a marginal OR). The third used mixed-effects models (for continuous outcomes) or GEEs with an exchangeable correlation structure (for binary outcomes, to ensure a marginal OR was estimated). The first two approaches (IEEs and analysis of cluster-level summaries) are unbiased for both the participant- and cluster-average estimands provided they are implemented using the correct weighting scheme,^8^ whereas the latter two models (mixed-effects models/GEEs with exchangeable correlation) can be biased for both estimands when there is informative cluster size.^8^ For each estimator, treatment arm was the only variable included in the model, and only participants with available outcome data were included in the model (see Table S1 in the Supplemental Material for the number of participants excluded for each outcome due to missing data).

We implemented IEEs for the participant-average effects using GEEs with an independence working correlation structure with cluster-robust standard errors and equal weight for each patient. IEEs for the cluster-average effect were implemented the same way, except that observations were weighted by $1/n_{i}$ , where $n_{i}$ denotes the size of the participant’s cluster. We calculated $n_{i}$ separately for each outcome, based on the number of participants within each cluster with available data. Mixed-effects models included a random intercept for cluster. GEEs with an exchangeable working correlation structure used cluster-robust standard errors and had equal weight for each patient.

For all GEE models (for both independence and exchangeable working correlation structures), we implemented the small-sample variance correction proposed by Mancl and DeRouen,^23^ and for mixed-effects models, we implemented the Kenward–Roger degree of freedom correction.^7,24^

To examine the expected variability between the cluster- and participant-average estimators when there was no informative cluster size, we conducted a small simulation study to explore this; details are in the Supplemental Material.

## Results

### Continuous outcomes

**Figure 1.**
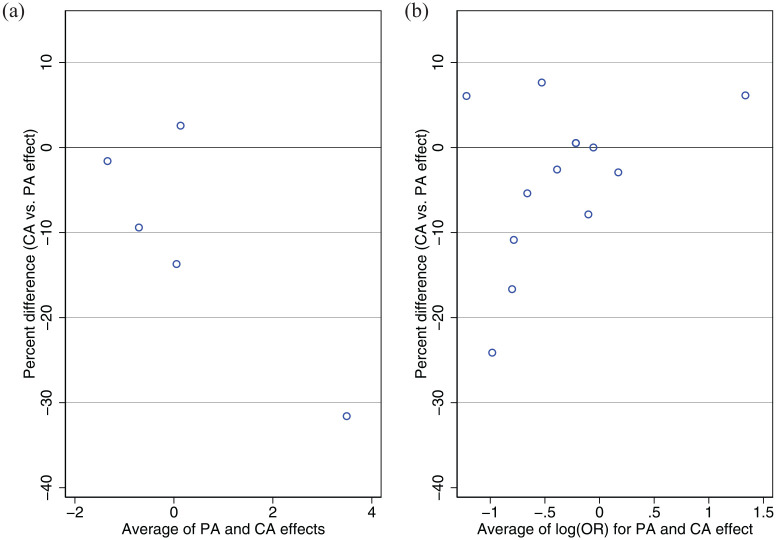
Bland–Altman plot of percent difference between cluster-average versus participant-average effects. (a) shows results for continuous outcomes and (b) shows results for binary outcomes. CA: cluster-average. PA: participant-average. OR: odds ratio.

Results are shown in Table 2 and Figure 1. There were moderate to large differences (>10% relative difference) between the participant- and cluster-average estimates (mean difference) for two of five (40%) outcomes. The most notable difference was for the EQ-5D VAS score, where the participant-average estimate was 4.15 (95% confidence interval (CI): −3.37 to 11.66) but was only 2.84 (95% CI: −7.37 to 13.04) for the cluster-average estimate, a reduction of 32%. Similarly, the mixed-effects model estimate for the EQ-5D VAS score differed by 22% (3.23, 95% CI: −6.70 to 13.16) from the participant-average effect.

**Table 2.** Difference in estimates from participant-average estimators versus cluster-average estimators and mixed-effects models for continuous outcomes. Treatment effects are differences in means (restrictive versus liberal transfusion strategy), presented with 95% CIs.

Outcome	Participant-average estimator (IEEs)^a^	Cluster-average estimator (IEEs)^b^	Mixed-model with random intercept	% difference cluster-average versus participant-average effect	% difference mixed-model versus participant-average effect
Number of days spent in hospital	−1.35 (−2.18, −0.53)	−1.33 (−2.31, −0.35)	−1.35 (−2.55, −0.15)	−1.6	0
EQ-5D	0.058 (−0.046, 0.162)	0.050 (−0.115, 0.215)	0.052 (−0.107, 0.212)	−13.7	−9.9
EQ-5D VAS score	4.15 (−3.37, 11.66)	2.84 (−7.37, 13.04)	3.23 (−6.70, 13.16)	−31.6	−22.0
Number red blood cell transfusions	−0.74 (−1.62, 0.14)	−0.67 (−1.60, 0.26)	−0.69 (−1.60, 0.22)	−9.4	−7.3
Average adherence	0.13 (0.08, 0.19)	0.14 (0.07, 0.21)	0.14 (0.07, 0.21)	2.6	1.7

CI: confidence interval; IEEs: independence estimating equations; EQ-5D: EuroQol-5D; VAS: visual analogue scale.

a Estimated using GEEs with an independence working correlation structure with cluster-robust standard errors and equal weight for each patient.

b Estimated using a linear regression model applied to unweighted cluster-level summaries.

### Binary outcomes

Results are shown in Table 3 and Figure 1. There were moderate to large differences (>10% relative difference) between the participant- and cluster-average estimates for 3 of 12 (25%) outcomes.

**Table 3.** Difference in estimates from participant-average estimators versus cluster-average estimators and GEEs with exchangeable correlation structures for binary outcomes. Treatment effects are marginal ORs (restrictive versus liberal transfusion strategy), presented with 95% CIs.

Outcome	Participant-average estimator (IEEs)^a^	Cluster-average estimator (IEEs)^b^	GEE with exchangeable correlation structure^c^	% difference cluster-average versus participant-average effect	% difference GEE versus participant-average effect
Further bleeding (day 28)	0.53 (0.21, 1.36)	0.50 (0.18, 1.43)	0.52 (0.20, 1.38)	−5.4	−1.7
Further bleeding (in-hospital)	0.48 (0.12, 2.01)	0.43 (0.10, 1.84)	0.45 (0.11, 1.89)	–10.9	−7.6
Mortality (in-hospital)^c^	0.80 (0.33, 1.97)	0.81 (0.30, 2.15)	0.80 (0.33, 1.97)	0.5	0.0
Thromboembolic/ischaemic events (day 28)	0.49 (0.16, 1.50)	0.41 (0.14, 1.21)	0.48 (0.16, 1.45)	−16.7	−2.4
Thromboembolic/ischaemic events (in-hospital)	0.43 (0.07, 2.48)	0.33 (0.06, 1.77)	0.41 (0.07, 2.36)	−24.1	–4.7
Infection (day 28)	0.94 (0.26, 3.39)	0.87 (0.25, 3.02)	0.88 (0.25, 3.06)	−7.9	–6.9
Infection (in-hospital)	0.95 (0.30, 2.99)	0.95 (0.32, 2.81)	0.95 (0.32, 2.83)	0.0	0.1
Acute transfusion reaction (in-hospital)	0.29 (0.09, 0.92)	0.30 (0.07, 1.40)	0.30 (0.13, 0.66)	6.1	2.8
Surgery/radiology (in-hospital)	1.21 (0.25, 5.85)	1.17 (0.25, 5.37	1.19 (0.25, 5.62)	–2.9	−1.1
Therapeutic endoscopy (in-hospital)	0.69 (0.34, 1.40)	0.67 (0.29, 1.54)	0.68 (0.31, 1.49)	–2.6	−1.4
At least one red blood cell transfusion	0.57 (0.22, 1.49)	0.61 (0.26, 1.44)	0.60 (0.25, 1.45)	7.6	6.0
Fully adhered to protocol	3.69 (1.51, 8.99)	3.92 (1.14, 13.44)	3.83 (1.25, 11.73)	6.1	3.7

GEEs: generalised estimating equations; CI: confidence interval; IEEs: independence estimating equations.

a Estimated using GEEs with an independence working correlation structure with cluster-robust standard errors and equal weight for each patient.

b Estimated using GEEs with an independence working correlation structure with cluster-robust standard errors and each patient weighted by $1/n_{i}$ (where $n_{i}$ denotes the cluster size).

c Estimated using GEEs with an exchangeable working correlation structure with cluster-robust standard errors and equal weight for each patient.

The most notable difference was for in-hospital thromboembolic or ischaemic events, where the participant-average OR was 0.43 (95% CI: 0.07 to 2.48) but was almost 25% lower at 0.33 (95% CI: 0.06 to 1.77) for the cluster-average OR. GEEs with an exchangeable correlation structure were similar to the IEE estimate of participant-average effect (odds ratio (OR): 0.41; 95% CI: 0.07 to 2.36).

### Simulation study

Full results are available in the Supplemental Material. Briefly, we found the probability of observing a difference as extreme as that of the EQ-5D VAS score if there was no informative cluster size was 19%. However, we found the probability of observing a greater than 10% difference between cluster- and participant-average estimates for 5 or more of the 17 outcomes (as observed in the TRIGGER trial) was only 4.8%.

## Discussion

In CRTs, the participant- and cluster-average treatment effects can differ when there is informative cluster size, and standard estimators such as mixed-effects models and GEEs with an exchangeable correlation structure can be biased. However, until now there has been, to our knowledge, no empirical assessment of informative cluster size in the context of a specific CRT. In this re-analysis of a previously published CRT, we aimed to explore whether informative cluster size may be present. For several outcomes, we identified notable differences between estimates for the participant- and cluster-average effects, as well as between IEEs and estimates affected by informative cluster size such as mixed-effects models or GEEs with an exchangeable correlation structure. For example, the treatment effect for the EQ-5D VAS score using a cluster-average estimator was 32% smaller than using a participant-average estimator (2.84 versus 4.15) and was 22% smaller using a mixed-effects model (3.23 versus 4.15). We hypothesise these differences may be due to informative cluster size.

These results highlight the need to formally consider the target estimand at the trial design stage (participant- versus cluster-average, and other aspects such as handling of intercurrent events, marginal versus cluster-specific, and so on) and choose an estimator that is aligned to that estimand. If informative cluster size occurs, then standard estimators such as mixed-effects models and GEEs with an exchangeable correlation structure may be biased, and methods such as IEEs or cluster-level summaries are required, as these estimators are unaffected by informative cluster size. Determining whether informative cluster size is likely may depend on various factors, such as type of population (e.g. how variable the type of participants and clusters are) and the type of intervention, variations in cluster size. Therefore, a case-by-case evaluation is required, which should be based on both a qualitative assessment using subject matter knowledge and a quantitative evidence from previous similar studies. While it is likely that some studies are not affected by informative cluster size, it should not be dismissed without careful thought when variable cluster sizes are anticipated at the study outset.

It should be noted that using IEEs or the analysis of cluster-level summaries may have implications for sample size, with potentially larger sample sizes required as compared with alternative approaches such as mixed-effects models or GEEs. Alternatively, if mixed-effects models or GEEs are used, IEEs and cluster-level summaries could be used as sensitivity analyses to explore whether inferences may be affected by informative cluster size. In practice, participant- and cluster-average effects will also need to be defined as either marginal or cluster-specific, and an estimator which allows for both aspects (e.g. marginal participant-average and cluster-specific cluster-average) will need to be chosen. Further guidance on choosing estimators which allow for both aspects of the estimand is available elsewhere.^22^

It may not in general be easy to identify whether informative cluster size is likely at the design stage. For instance, in the TRIGGER trial, there was no reason to suspect informative cluster size would occur when designing the trial. Further complicating the task, it appears that informative cluster size can occur for some outcomes but not others, requiring investigators to make this judgement not for the trial as a whole, but for each outcome separately. One setting where an impact of informative cluster size can be confidently ruled out is when there is little to no variation in the cluster size. In this case, mixed-effects models and GEEs with an exchangeable correlation structure will not be biased; however, there will be little gain in efficiency from these models compared with IEEs in this setting.^20,25^

Some investigators may feel that potential bias from informative cluster size is of less concern than statistical efficiency, given common problems around the number of available clusters and challenges in patient recruitment. Thus, they may argue that a small amount of bias from mixed-effects models or GEEs with an exchangeable correlation structure is worth it if it leads to a substantial reduction in the required sample size, or a corresponding increase in statistical power. While we acknowledge the logic behind this viewpoint, we argue that its general application may be challenging for two reasons. First, both efficiency and potential bias from informative cluster size will be driven by the size of the intraclass correlation coefficient, indicating that the situations where mixed-effects models/GEEs with an exchangeable correlation structure can provide substantial gains in efficiency may be the same situations where they are be prone to extreme bias. Second, because the bias from informative cluster size can be in either direction, the gains in efficiency from mixed-effects models/GEEs with an exchangeable correlation structure may be offset by a possible reduction in the size of estimated treatment effects (i.e. downwards bias), and so these methods may not lead to power gains compared with IEEs. Future simulation studies to compare metrics like power and precision between mixed-effects models/GEEs with an exchangeable correlation and IEEs both under informative cluster size and no informative cluster size are warranted to more thoroughly evaluate the bias/variance trade-off between these approaches.

A limitation of this study is that there is currently no formal test to identify informative cluster size. Thus, we were not able to differentiate to what extent differences between estimators were due to informative cluster size compared with random variation. If differences were simply due to random variation, we would expect these to occur in either direction. However, large differences of >10% occurred in only one direction (all were negative, denoting cluster-average effects were smaller than participant-average effects), and this consistency of effect lends credence to the theory they may be due to informative cluster size. A small simulation study (Supplemental Material) confirmed these results were unlikely to be due to chance, though could not rule it out entirely. An alternative approach to evaluate informative cluster size could be to try and model the association between cluster size and outcomes/treatment effects as an indicator for informative cluster size; however, this approach relies on specifying the correct functional form between cluster size and outcomes, which is challenging, and gives little indication as to the impact of potential informative cluster size on results. As such, in our view, directly comparing the two estimates (participant- versus cluster-average) provides a simpler indication as to whether informative cluster size is a concern. Second, for some outcomes, it may be possible that informative cluster size was actually induced by missing data (for instance, if a large proportion of participants with good outcomes were missing in some clusters but not others, this may make smaller clusters appear to have worse outcomes than larger clusters). In this case, informative cluster size is actually a missing data problem, requiring an appropriate missing data approach. Of the five outcomes with >10% differences, two had <1% missing data, one had 6% missing, and two had 46% missing (the two EQ-5D measures); thus, given the high rate of missingness, we cannot rule out that informative cluster size for the two EQ-5D measures was in fact an artefact of the missing data. Finally, our re-analysis was limited to a single trial, so although we hypothesise that informative cluster size did occur, we cannot say how frequently it may be a concern in practice.

Our results suggest several areas for future research. First, re-analyses of other CRTs would be useful to determine whether the results found here are unique or not. Second, establishing empirical evidence of in what context informative cluster size is likely would be helpful to aid in planning of future CRTs. Third, though sample size calculations are available for IEEs,^20,25^ these may need to be adapted when informative cluster size is anticipated.

## Conclusion

In this re-analysis, we found that estimates from different estimators could differ markedly for some outcomes, which may be due to the presence of informative cluster size. Careful consideration of the estimand and the plausibility of assumptions underpinning each estimator can help ensure an appropriate analysis method is used. IEEs and the analysis of cluster-level summaries (with appropriate weighting for each to correspond to either the participant-average or cluster-average treatment effect) are a desirable choice when informative cluster size is deemed possible, due to their unbiasedness in this setting.

## Supplemental Material

sj-docx-1-ctj-10.1177_17407745231186094 – Supplemental material for Informative cluster size in cluster-randomised trials: A case study from the TRIGGER trial Supplemental material, sj-docx-1-ctj-10.1177_17407745231186094 for Informative cluster size in cluster-randomised trials: A case study from the TRIGGER trial by Brennan C Kahan, Fan Li, Bryan Blette, Vipul Jairath, Andrew Copas and Michael Harhay in Clinical Trials
