# Supplementary material for: Informative cluster size in cluster-randomised trials: A case study from the TRIGGER trial
Source: Clin Trials. 2023 Jul 13;20(6):661–9. doi: 10.1177/17407745231186094 (PMC10638852; doi:10.1177/17407745231186094)
Supplement: sj-docx-1-ctj-10.1177_17407745231186094 – Supplemental material for Informative cluster size in cluster-randomised trials: A case study from the TRIGGER trial [file sj-docx-1-ctj-10.1177_17407745231186094.docx]

**Supplementary appendix - *Informative cluster size in cluster-randomised trials: A case study from the TRIGGER trial***

**Table S1 – Number of participants with missing outcome data excluded from each analysis.** Data are No. (%).

|  |  | **Number of participants with available data in each cluster** | | | | | |
| --- | --- | --- | --- | --- | --- | --- | --- |
| **Outcome** | **Number missing data (N=936)** | **Cluster 1** | **Cluster 2** | **Cluster 3** | **Cluster 4** | **Cluster 5** | **Cluster 6** |
| Time to hospital discharge | 35 (4) | 183 | 90 | 107 | 181 | 195 | 145 |
| EQ-5D | 432 (46) | 84 | 63 | 69 | 61 | 135 | 92 |
| EQ-5D VAS score | 433 (46) | 83 | 63 | 69 | 61 | 134 | 93 |
| Number red blood cell transfusions | 1 (<1) | 187 | 91 | 111 | 191 | 201 | 154 |
| Average adherence | 1 (<1) |  |  |  |  |  |  |
| Further bleeding (day 28) | 31 (3) | 184 | 89 | 109 | 181 | 195 | 147 |
| Further bleeding (in-hospital) | 1 (<1) | 187 | 91 | 111 | 191 | 201 | 154 |
| Mortality (in-hospital) | 2 (<1) | 187 | 91 | 111 | 191 | 201 | 153 |
| Thromboembolic/ischemic events (day 28) | 53 (6) | 180 | 88 | 105 | 176 | 193 | 141 |
| Thromboembolic/ischemic events (in-hospital) | 1 (<1) | 187 | 91 | 111 | 191 | 201 | 154 |
| Infection (day 28) | 269 (29) | 121 | 75 | 78 | 134 | 150 | 109 |
| Infection (in-hospital) | 2 (<1) | 187 | 91 | 111 | 191 | 201 | 153 |
| Acute transfusion reaction (in-hospital) | 12 (1) | 184 | 90 | 111 | 190 | 199 | 150 |
| Surgery/radiology (in-hospital) | 1 (<1) | 187 | 91 | 111 | 191 | 201 | 154 |
| Therapeutic endoscopy (in-hospital) | 1 (<1) | 187 | 91 | 111 | 191 | 201 | 154 |
| At least one RBC transfusion | 1 (<1) | 187 | 91 | 111 | 191 | 201 | 154 |
| Fully adhered to protocol | 1 (<1) | 187 | 91 | 111 | 191 | 201 | 154 |

**Simulation study to evaluate expected variability between the cluster- and participant-average estimators when there is no ICS**

***Methods***

We performed two simulations to help provide context to our observed results. The first simulation was to determine how frequently we would expect to observe extreme differences in the % difference between the cluster- and participant-average estimator when there is no ICS. The second simulation was to determine how frequently we would expect to observe 5 or more of the 17 outcomes examined showing >10% difference, all in the same direction, if there was no ICS (as this is what we observed in our re-analysis).

For the first simulation, we generated 10,000 datasets based on the TRIGGER trial (i.e. we used 6 clusters, 3 assigned to each treatment, used the number of participants with observed outcome data in each cluster, and used the observed within- and between-cluster variances from the trial). We based simulations on the outcome EQ-5D VAS score. We set the between-cluster variance to 6.2, and the residual variance to 463.1 (denoting an intraclass correlation coefficient of 0.013). We set the treatment effect to 4.15; because we simulated data under the assumption there was ICS, this implies the participant- and cluster-average treatment effects are both 4.15. For each dataset, we implemented the cluster- and participant-average estimators in the same way as for the re-analysis, and then computed the % difference. We then tabulated the percentage of datasets for which the % difference was greater than in absolute value the % difference observed in our re-analysis (31.6%).

For the second simulation, we generated 10,000 datasets, each of which contained 17 outcomes. We did not generate the outcomes themselves, but instead generated whether each outcome had a >10% (absolute) difference between the participant- and cluster-average estimator in that dataset, using the probability of such a difference calculated from the 1^st^ simulation study (this probability was calculated as 22.5% in either direction, i.e. there was a 22.5% chance of a >10% negative difference, and a 22.% chance of a >10% positive difference). Hence, each outcome could show (i) a <-10% difference; (ii) a >10% difference; or (iii) no difference (i.e. less than 10% absolute difference). We then tabulated the percentage of datasets for which 5 or more outcomes showed a >10% absolute difference in the same direction, with no outcomes showing a >10% absolute difference in the opposite direction.

***Results***

For the first simulation study, we found the probability of observing a % difference as more extreme than that observed in our re-analysis of the EQ-5D VAS score, if there was no ICS, was 19%.

For the second simulation study, we found the probability of observing 5 or more outcomes showing a >10% absolute difference, all in the same direction with no outcomes showing such a difference in the opposite direction, if there was no ICS, was 4.8%.

***Conclusions***

These results suggest that the results observed in our re-analysis were unlikely to occur if there was truly no ICS in TRIGGER (i.e. had a less than 1 in 20 chance of occurring), but were not impossible. Hence, we cannot say for sure whether or not there is ICS in TRIGGER. We note that these results should be interpreted cautiously, as they rely on the specific manner of generating the data (i.e. that we have modelled the “true” data generating process, which is unknown).

**Stata code for simulation study 1**

qui egen size = count(centre), by(centre)

egen pickone = tag(centre)

tab size treat if pickone == 1

set seed 19991

local reps = 10000

cap postclose mysim

postfile mysim ///

///

i ///

trt_eff_PA_IEE_indlevel ci_ll_PA_IEE_indlevel ci_ul_PA_IEE_indlevel ///

pvalue_PA_IEE_indlevel ///

///

trt_eff_CA_IEE_indlevel ci_ll_CA_IEE_indlevel ci_ul_CA_IEE_indlevel ///

pvalue_CA_IEE_indlevel ///

///

percent_diff_CA_vs_PA ///

///

using "file", replace

forval i = 1/`reps' {

dis in red "`i'"

qui{

clear

set obs 6

gen centre = _n

gen size = 61 if centre == 1

replace size = 63 if centre == 2

replace size = 69 if centre == 3

replace size = 83 if centre == 4

replace size = 93 if centre == 5

replace size = 134 if centre == 6

gen unif = runiform()

sort unif

gen new_id = _n

gen z = 0 if new_id <=3

replace z = 1 if z != 0

gen u = rnormal(0, sqrt(6.2))

expand size

gen e = rnormal(0, sqrt(463.1))

gen y = 0 + 4.15*z + u + e

qui reg y z, vce(cluster centre)

matrix analysis_results = r(table)

local trt_eff_PA_IEE_indlevel = analysis_results[1,1]

local ci_ll_PA_IEE_indlevel = analysis_results[5,1]

local ci_ul_PA_IEE_indlevel = analysis_results[6,1]

local pvalue_PA_IEE_indlevel = analysis_results[4,1]

qui reg y z [pw=1/size], vce(cluster centre)

matrix analysis_results = r(table)

local trt_eff_CA_IEE_indlevel = analysis_results[1,1]

local ci_ll_CA_IEE_indlevel = analysis_results[5,1]

local ci_ul_CA_IEE_indlevel = analysis_results[6,1]

local pvalue_CA_IEE_indlevel = analysis_results[4,1]

local percent_diff_CA_vs_PA = 100*(`trt_eff_CA_IEE_indlevel'/`trt_eff_PA_IEE_indlevel' - 1)

}

post mysim ///

///

(`i') ///

(`trt_eff_PA_IEE_indlevel') (`ci_ll_PA_IEE_indlevel') (`ci_ul_PA_IEE_indlevel') ///

(`pvalue_PA_IEE_indlevel') ///

///

(`trt_eff_CA_IEE_indlevel') (`ci_ll_CA_IEE_indlevel') (`ci_ul_CA_IEE_indlevel') ///

(`pvalue_CA_IEE_indlevel') ///

///

(`percent_diff_CA_vs_PA')

}

postclose mysim

use "file", clear

summ percent_diff_CA_vs_PA, d

gen new_percent_diff = abs(percent_diff_CA_vs_PA)

sort new_percent_diff

dis 1 - 8111/10000

**Stata code for simulation study 2**

local prob_extreme = 0.225

set seed 6777

local reps = 10000

cap postclose mysim

postfile mysim ///

///

i ///

yes ///

///

using "file", replace

forval i = 1/`reps' {

qui {

clear

local prob_extreme = 0.225

set obs 17

gen id = 1

gen tp = _n

gen unif = runiform()

gen result = 0 if unif < `prob_extreme'

replace result = 1 if unif >= `prob_extreme' & unif < (1-`prob_extreme')

replace result = 2 if unif >= (1-`prob_extreme')

drop unif

reshape wide result, i(id) j(tp)

egen n_extreme_up = anycount(result*), val(2)

egen n_extreme_down = anycount(result*), val(0)

gen extreme5 = 1 if n_extreme_up >= 5 & n_extreme_down == 0

replace extreme5 = 1 if n_extreme_up == 5 & n_extreme_down >= 5

replace extreme5 = 0 if extreme5 != 1

local yes = extreme5[1]

}

post mysim ///

///

(`i') ///

(`yes')

}

postclose mysim

use "file", clear

tab yes
